# Longitudinal Intravital Microscopy Reveals Axon Degeneration Concomitant With Inflammatory Cell Infiltration in an LPC Model of Demyelination

**DOI:** 10.3389/fncel.2020.00165

**Published:** 2020-06-23

**Authors:** Bilal El Waly, Emeline Buttigieg, Cem Karakus, Sophie Brustlein, Franck Debarbieux

**Affiliations:** ^1^Institut des Neurosciences de la Timone, UMR 7289, CNRS, Aix-Marseille Université, Marseille, France; ^2^Centre Européen de Recherche en Imagerie Médicale, Aix-Marseille Université, Marseille, France; ^3^Molecular Physiology, Center for Integrative Physiology and Molecular Medicine, University of Saarland, Homburg, Germany; ^4^CNRS, IBDM, Turing Center for Living System, Aix-Marseille Université, Marseille, France

**Keywords:** lysophosphatidylcholine, demyelination, neurodegeneration innate immune cells, CARS, spinal glass window

## Abstract

Demyelination and axon degeneration are major events in all neurodegenerative diseases, including multiple sclerosis. Intoxication of oligodendrocytes with lysophosphatidylcholine (LPC) is often used as a selective model of focal and reversible demyelination thought to have no incidence for neurons. To characterize the cascade of cellular events involved in LPC-induced demyelination, we have combined intravital coherent antistoke Raman scattering microscopy with intravital two-photon fluorescence microscopy in multicolor transgenic reporter mice. Moreover, taking advantage of a unique technique of spinal glass window implantation, we here provide the first longitudinal description of cell dynamics in the same volume of interest over weeks after insults. We have detected several patterns of axon–myelin interactions and classified them in early and advanced events. Unexpectedly, we have found that oligodendrocyte damages are followed by axon degeneration within 2 days after LPC incubation, and this degeneration is amplified after the recruitment of the peripheral proinflammatory cells at day 4. Beyond day 7, the recovery of axon number and myelin takes 3 more weeks postlesion and involves a new wave of anti-inflammatory innate immune cells at day 14. Therefore, recurrent imaging over several weeks suggests an important role of peripheral immune cells in regulating both the axonal and oligodendroglial fates and thereby the remyelination status. Better understanding the recruitment of peripheral immune cells during demyelinating events should help to improve diagnosis and therapy.

## Introduction

Myelin sheath, generated by oligodendrocytes (OLs) in the central nervous system (CNS), is a multilayer membrane wrapping axons and providing electrical insulation, high-speed axonal conduction, and trophic support (Kaplan et al., 1997). Demyelination that occurs in most neurodegenerative diseases is due to the pathological destruction of myelin sheaths and subsequent degeneration of myelinating OLs. Various models of demyelination have been established in rodents, among which oligodendroglial intoxication by lysophosphatidylcholine (LPC) is the most common (El Waly et al., 2014).

Lysophosphatidylcholine is a glycerophospholipid naturally occurring in all cell membranes that is particularly enriched in the CNS white matter. Generated through the hydrolysis of membrane plasmalogens by phospholipase A_2_ (Kougias et al., 2006), it can also be supplemented directly from emulsifier-rich food (Shah et al., 2017). LPC plasmatic concentration must be regulated physiologically because 100-μM doses already turn out toxic for most cell types, from OLs (Plemel et al., 2018) to endothelial cells (Akerele and Cheema, 2015). Endogenous LPC is indeed likely involved in atherosclerosis (Chang et al., 2017) and affects the occurrence of major depressive disorders (Liu et al., 2016).

At the cellular level, low-dose 1% LPC (∼10 μM) has been reported to trigger the selective death of spinal OLs but not the death of axons (Hall, 1972). Seven days after exposure, demyelination reaches a peak that is followed by a remyelination process of axons close to completion within 4 weeks after exposure (Jeffery and Blakemore, 1995). Despite its lack of direct effect on axons, LPC injection was reported to trigger inflammation and subsequent secondary Wallerian degeneration characterized by dystrophic axonal retraction bulb at the injury site and discontinuous spherical debris along their distal part (Ousman and David, 2000, 2001). From these experiments, it remains, however, unclear whether LPC itself generates an inflammatory environment because of the release of damage-associated molecular patterns from permeabilized OLs or because its injection pipette induced mechanical destruction of the parenchyma.

To address this question, we have thus established a unique murine model of focal LPC incubation that does not sever axons mechanically. Using a combination of intravital two-photon microscopy and coherent antistoke Raman scattering (CARS) microscopy, we have then simultaneously obtained access in real-time to the label-free detection of myelin (Shi et al., 2011), as well as the fate of fluorescent axonal networks in the presence of recruited inflammatory cells (Fenrich et al., 2013b). Taking advantage of the unique glass window protocol developed in our laboratory, we have thus obtained a highly time-resolved description of LysM^+^ myeloid cell contribution to axons and myelin reorganization. We report for the first time that initial intoxication of OLs is itself responsible for the early recruitment of proinflammatory immune cell coincident with the onset of Wallerian degeneration. Soon after, it, however, converts into a prohealing inflammation that is coincident with remyelination and prior to the delayed increase of fully remyelinated axons.

## Materials and Methods

### Mice

All experimental and surgical protocols were performed following the guidelines established by the French Ministry of Agriculture (Animal Rights Division). The architecture and functioning rules of our animal house, as well as our experimental procedures, have been approved by the “Direction Départementale des Services Vétérinaires” and the ethic committee (ID numbers #18555-2019011618384934 and A1305532 for animal house and research project, respectively).

Seven- to Ten-week-old fluorescent reporter mice were used for spinal cord glass window implantation (Fenrich et al., 2013a) and LPC demyelination. Thy1–CFP and/or Thy1–CFP//LysM-EGFP//Cd11C-EYFP triple-heterozygous transgenic mice were used.

### Spinal Cord Glass Window Implantation and LPC Model of Demyelination

**FIGURE 1:**
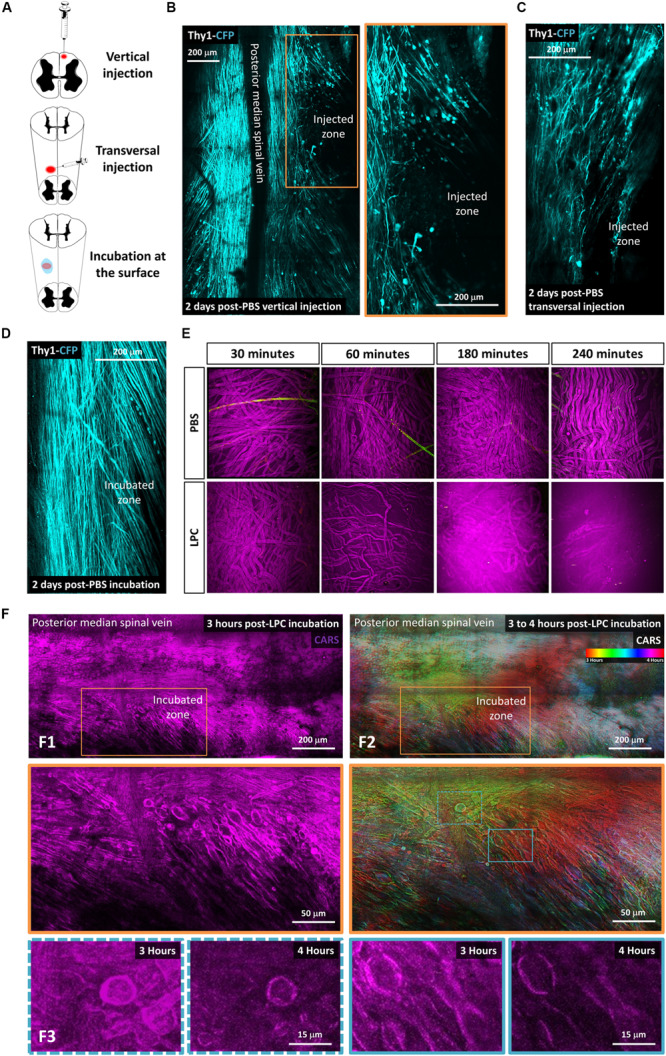
Lysophosphatidylcholine incubation on the spinal cord surface avoids artifactual mechanical insult to axons. **(A)** Schematic description of the three different ways tested to make LPC model of demyelination: vertical injection, transversal injection, and superficial incubation. **(B–D)** *In vivo* biphoton acquisition through the dorsal implanted windows showing the dorsal Thy1–CFP axon network 2 days after vertical injection of PBS, transversal injection of PBS, and superficial PBS incubation, respectively. **(E)** *In vitro* incubation of either PBS or LPC 1% on the surface of sciatic nerve for 30 min, 1 h, 4 h, and 5 h. **(F)** *In vivo* biphoton acquisition through the implanted dorsal windows showing F1: CARS signal (magenta) starting 3 h after LPC incubation at low (top) and high magnification (bottom). F2: Average intensity projection of time-coded color images highlighting the evolution of myelin degradation over time at low (top) and high magnification (bottom). F3: CARS signals in the regions of interest outlined in F2 (bottom) are represented at two different times points, 3 and 4 h, respectively.

The window was applied as described in Fenrich et al. (2012) study. Prior to glass window sealing, the dura mater was opened locally to directly expose the dorsal white matter to 1% LPC (Sigma, L1381) in 0.9% NaCl that we incubated for 1 h (Figure 1).

### Biphoton and CARS Microscopy Methods

Coherent antistoke Raman scattering imaging was done using OPO femtosecond laser source (80 MHz 150 fs; Coherent, Santa Clara, CA, United States) with excitation wavelengths λp = 806 and λs = 1,050 nm, temporally synchronized and spatially overlapped on the sample plane. The corresponding excited resonances correspond to lipid vibrations approximately 2,850 cm^–1^ with a bandwidth 150 cm^–1^, dominated by the CH2 and CH3 stretching modes. Whereas nonlinear excitations of CFP fluorescence and EYFP fluorescence were optimal at λp = 806 and λs = 1,050 nm, respectively, wavelengths mixing between λp and λs provided the excitation of EGFP (Ricard et al., 2016). The CARS intensity was determined based on day 0 (D0); indeed, all our acquisitions were made in the same way and using the same CARS intensity.

### Cells Dissociation, Sorting, and Quantitative PCR

Spinal cords were extracted 4, 7, or 14 days after LPC incubation. Spinal cords were dissected manually. Cells were dissociated using the MACs Adult Brain Dissociation Kit, mouse and rat (Miltenyi Biotec, Paris, France). GFP^+^ cells were sorted using FACS (fluorescence-activated cell sorting) into lysis buffer (10:1 mix of Resuspension Buffer and Lysis Enhancer from Cells Direct one-step quantitative reverse transcription–polymerase chain reaction (RT-PCR) kit; Thermo Fisher Scientific, Waltham, MA, United States). Reverse transcription reactions were performed using 5 μg total RNA. Polymerase chain reaction reactions were performed in 20 μL of Superscript II reaction buffer (Invitrogen, Carlsbad, CA, United States) containing 0.01 M dithiothreitol, 7.5 ng/μL of dN6, 20 U of RNase inhibitor (Invitrogen), 10 mM dNTP and 200 U of Superscript II reverse transcriptase (Invitrogen) for 1 h at 42°C. Real-time PCR reactions on cDNA were performed using the LightCycler 480 system (Roche, Indianapolis, IN, United States) using the SYBR Green I Master Kit (Eurogentec, Seraing, Belgium) with 2 μL of cDNA and 200 nM of each PCR primer. Each reaction was performed in triplicate.

### Immunolabeling on Spinal Cord Sections

Mice were transcardially perfused with 4% paraformaldehyde. The spinal cords were removed, postfixed overnight, and cut into 100-μm coronal and sagittal sections using a vibratome (Leica Microsysteme, Rueil-Malmaison, France). Immunofluorescent labeling was performed on sections fixed with paraformaldehyde 4%. The following antibody was used: anti-MBP (rat, 1/500; BioRad MCA409S, CA, United States). The sections and cells were incubated with appropriate Alexa-conjugated secondary antibodies and then counterstained with Hoechst 33258 (1/1,000; Sigma-Aldrich, St. Louis, MI, United States).

### Image Analysis, Quantification, and Statistical Analysis

Images were handled using ZEN 2.1 (Zeiss, Oberkochen, Germany) and ImageJ software (ImageJ, NIH, United States). Axons and cells were counted manually. All the presented values are means ± SEM unless otherwise stated. Data were statistically processed with nonparametric Mann–Whitney *U* tests for independent two-group comparison.

## Results

### Development of LPC Model of Focal Demyelination in the Spinal Cord Without Mechanically Induced Axonal Damages

Our aim was to take advantage of innovative intravital imaging modalities to describe the direct and indirect effects of LPC on spinal myelin and axons. In earlier studies (Jeffery and Blakemore, 1995), the vertical needle used to inject LPC in the spinal cord likely produced significant Wallerian degeneration, which occurs even in absence of chemical injection (Dray et al., 2009; Fenrich et al., 2013b). Therefore, we first tried to minimize nonchemical contaminating sources of axonal death in this focal demyelination protocol by changing the penetration angle of the injection pipette from vertical to transversal (Figure 1). Two days after phosphate-buffered saline (PBS) 1 × injection (0.7 μL), we then compared the densities of suffering axons in these two conditions based on the presence of retraction bulbs, spherical debris, or wavy morphologies that precede Wallerian degeneration. Although transversal injection improved axonal sparing, PBS delivery through the capillary still resulted into significant axonal degeneration (31% ± 3%, *n* = 3 in transversal injection, vs. 57% ± 4.0%, *n* = 3 in vertical injection; Figure 1C). Noteworthy, this degeneration was totally avoided when replacing the intraparenchymal injection by PBS incubation of the spinal cord surface following the calibrated opening of the dura mater. One-hour PBS incubation left axons virtually undisturbed (3% ± 1.5%, *n* = 3), and axonal networks remained stable after 2 days (3.9% ± 0.7%, *n* = 3, Figure 1D).

Taking advantage of a simple *in vitro* sciatic nerve preparation, we next established the incubation conditions required to visibly destabilize the myelin sheath. Fresh sciatic nerve slices were incubated in 1% LPC for 30 min to 4 h prior to paraformaldehyde fixation and subsequent imaging by CARS microscopy. Coherent antistoke Raman spectroscopic microscopy is a nonlinear imaging technique revealing the endogenous contrast of lipids based on the vibrational signature of their CH2 bond. The high density of phospholipid chains in myelin sheath is responsible for a significant CARS contrast compared to unmyelinated regions of the CNS tissue. Unhealthy ruffling of myelin sheath was thus observed on 6% of axons after 30-min incubation. It was observed in 85% of axons following a 60-min incubation, and myelin was finally completely destroyed after 3- to 4-h incubation (Figure 1E).

A 1-h incubation time was then chosen to conduct intravital experiments in the spinal cord. This exposition produced detectable effect while limiting the overall surgery time prior to the final glass window implantation and the subsequent longitudinal imaging. In brief, LPC 1% was rinsed with PBS after 1 h, and the glass window was sealed before time lapse imaging of the lesion site over 4 h. Our focal incubation model conclusively triggered demyelination over a region that progressively spread to cover a surface that doubled over time (192% ± 13% *n* = 4) (Figure 1F) and reaching a depth of 156 μm (±18 μm) below the surface. As highlighted in Figure 1F2, lesion surrounding whitish myelin represents the stable myelin whose density is conserved in all time-coded color images. Red areas represent the initial demyelination zone, where myelin was present in the initial images but had disappeared at the time coded in green and blue. A second wave of degeneration was obvious in the green areas were myelin density had strongly declined at the time of blue–pink coding of the images. Because the intensity of CARS signal correlated almost perfectly with the level of MBP labeling on postmortem immunohistological staining (Supplementary Figure S1), we concluded that the combination of glass window and CARS imaging was ideal to follow the evolution of myelin degradation over time *in vivo*.

### Demyelination-Induced Wallerian Degeneration Following LPC Intoxication

**FIGURE 2:**
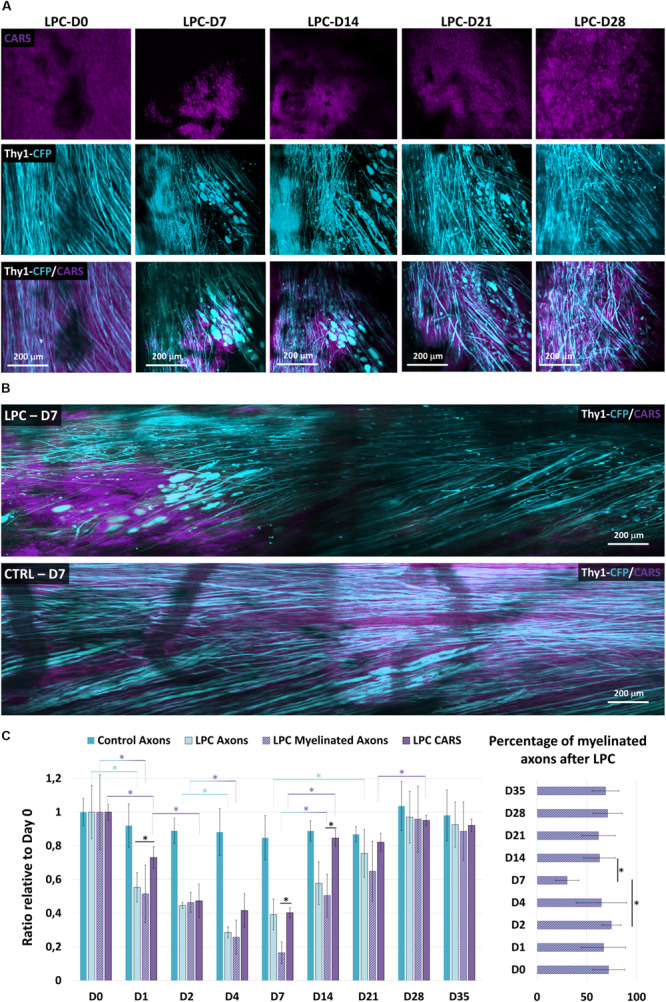
Longitudinal imaging reveals LPC-mediated myelin and axon degenerations and rescue. **(A)** Follow-up of the same LPC incubated zone through the dorsal implanted windows showing myelin content at days 0, 7, 14, 21, and 28 as detected with CARS in myelin sheath (magenta, top) and Thy1–CFP fluorescence (cyan, middle) and overlay (middle). **(B)** Intravital images of dorsal spinal cord 7 days following incubation of LPC or PBS. Note the localized disappearance of CARS signal and of axons selectively for LPC incubation. But not PBS. **(C)** Bar graphs presenting the evolution of the axonal density and the CARS signal both normalized to their value on the first day at days 0, 1, 2, 4, 7, 14, 21, 28, and 35 (we used at least five mice for each condition and for each time; total number *n* = 22). Bar graph showing the evolution of the percentage of myelinated axons over total axons in the receiver operating characteristic curve on days 0, 1, 2, 4, 7, 14, 21, 28, and 35 after LPC incubation. Asterisk indicate statistical significance *p* < 0.05.

The average CARS signal in a region of interest reported the lipid density irrespective of their distribution into structured myelin sheath or into degenerative myelosomes (Gasecka et al., 2017). The long-term consequences of a 1-h exposition to LPC were next characterized over weeks by 2 Photon-CARS microscopy. The same volume of interest was repeatedly acquired on the same animal at various postincubation times, and we compared the evolution of myelin coverage, with regard to the changes of fluorescent axon densities (Figure 2). Whereas 100% of Thy1–CFP axons are usually myelinated in the dorsal spinal cord region, LPC incubation immediately triggered demyelination that further extended during the following days. On D4, CARS intensity reached a minimal value that represented 42% of the initial intensity (Figure 2C). After quantifying the Thy1–CFP axons in a volume of interest representing 400 by 400 μm over a depth of 100 μm, we found that LPC exposure also triggered a brutal axonal degeneration in the first 2 days that left only 44% of the initial axonal density on D2. This initial loss of axons was followed by a second wave of degeneration that brought axonal density to 28% of its initial value on D4 (Figure 2C). Axonal degeneration was thus faster and larger than the demyelinating events highlighted by CARS and resulting either from the loss of myelin coverage or the clearance of myelin debris.

Conversely, axons regenerated linearly from D4 to D14, although CARS signal remained stuck at its minimal value in the first week and then promptly recovered during the second week (Figure 2C). On D14, however, axonal density represented only 57% of its initial value, whereas CARS signal exhibited an 80% recovery. Both myelin and axons were finally fully recovered by D28 (Figure 2C). The delay between the two phenomena suggested that the fate of neuronal and oligodendroglial networks is regulated by independent mechanisms. This idea was further supported by the observation of the delayed remyelination of axons that were already regenerated at D7 (Supplementary Figures S2A,B).

### Sequential Disruption of Myelin Sheath and Delayed Degeneration of Axons

**FIGURE 3:**
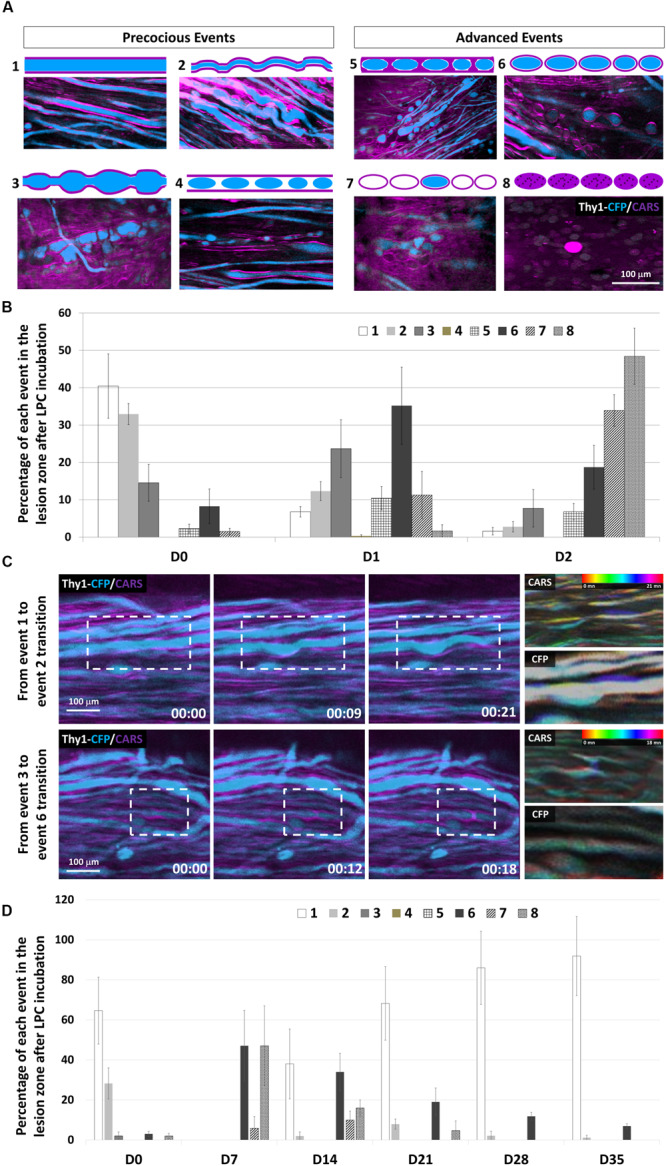
Subcellular effects of LPC incubation on myelin sheath and axons classified according to eight canonical features. **(A)** Intravital images and schematic representations of typical morphological features encountered in the lesion area. CARS signals (myelin, magenta) and Thy1–CFP fluorescence (axon, cyan). **(B)** Graph showing the relative occurrence of each event numbered from 1 to 8 in the lesion zone at days 0, 1, and 2 after LPC incubation (*n* = 5 mice for each time). **(C)** Outlining intravital time lapse images showing the transitions from event 1 to event 2 (top) and from event 3 to event 6 (Bottom). For each transition, temporal-color coding of the CARS channel and its corresponding CFP channel showing the subcellular morphological evolutions. **(D)** Graph showing the percentage of each event from 1 to 8 in the lesion zone at days 0, 7, 14, 21, 28, and 35 after LPC incubation (we used at least five mice for each condition and for each time; total number *n* = 13).

To clarify the sequence of cellular events responsible for the brutal loss of axons despite a progressive decline of CARS signal, we next looked for subtle changes of myelinated axon morphologies as readouts of the effect of LPC on OLs and their axonal counterpart. Eight typical morphological patterns of degeneration were established from the pool of images acquired from five animals (Figure 3A). (1) Both axon and myelin were straight and smooth, two markers of cellular health; (2) axon and surrounding myelin presented a wavy appearance; (3) axon and myelin formed bubble-like swellings; (4) disconnected axonal bubbles inside apparently healthy myelin sheath; (5) small axonal bubbles surrounded with myelin sheath; (6) discontinuous axon bubbles surrounded by myelin sheath; (7) pure myelin debris interleaved with mixed myelin/axons debris; and (8) debris of mixed composition. Immediately after LPC incubation, approximately 33% of axons exhibited a pathological spring-like wavy shape that suggested the likely occurrence of mechanical constraints on otherwise linear healthy axons sheaths (Figure 3A2); 14.5% of axons presented neuronal swelling apparently resulting from presumed lipidic constrictions (Figure 3A3). One day later, the incidence of spring-like shapes declined in favor of neuronal swelling (Figure 3A3), whereas Wallerian-like features (Figure 3A6) became the most represented pattern (Figure 3B). Wallerian degeneration further proceeded until D2 when fluorescent degenerative axonal bodies became sparser (Figures 3A7,B) and when large areas were covered with high background CARS signal overlaid with scattered nonfluorescent liposomes (Figure 3A8). The set of morphological patterns mainly observed in the earliest postincubation hours progressively evolved toward a set of patterns mainly observed on the second day postincubation (Figures 3A,B) as expected if the described degenerative stages in fact resulted from a morphological continuum of axonal shapes driven by biomechanical constraints.

Time-lapse images over hours conclusively demonstrated the possible evolution from one type of pattern to the next (Figure 3C), suggesting a gradual remodeling. Time-coded images of myelin and axons, respectively, highlighted a distortion of the myelin sheath that subsequently induced axonal deformation.

Most axons were in advanced degenerated states (Figures 3A6–8) by D2 (Figure 3B), a time when the background CARS signal was high despite the sparseness of structured myelin sheath segments (Figure 3A). Such degenerative events remained predominant until D7 when they started to decline massively, leaving space to the progressive recolonization by healthy axons (Figure 3D). Our results altogether support that early disorganization of myelin structure could trigger axonal fragmentation and neurodegeneration. Axonal recovery finally occurs even before the remyelination process starts.

### Inflammatory Cell Infiltration Concomitant With Axon Degeneration in LPC Model

**FIGURE 4:**
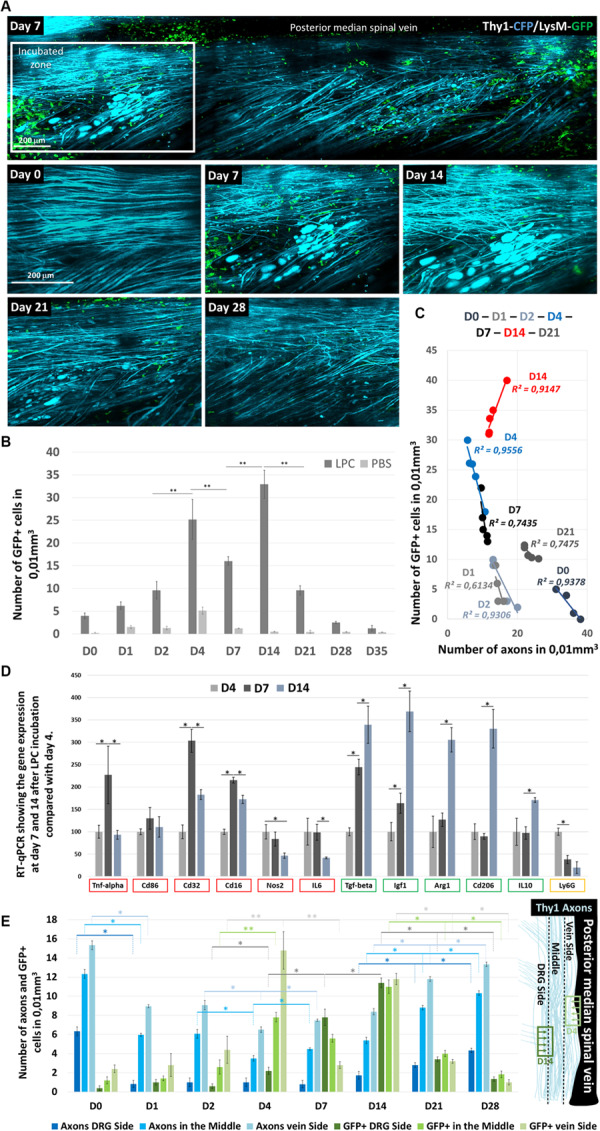
LysM-GFP^+^ cell infiltration in the Spinal cord and their contribution to axon degeneration. **(A)** Follow-up of the same zone showing LysM-GFP^+^ cells (green) and Thy1–CFP axons (cyan) at days 0, 7, 14, 21, and 28 after LPC incubation **(B)**. Graph comparing the number of GFP^+^ cells infiltrating the spinal cord at days 0, 1, 2, 4, 7, 14, 21, 28, and 35 after LPC or PBS incubation (*n* = 5 mice for each time and each condition). **(C)** Graph showing the correlation between the number of axons counted in a given mouse and the number of GFP^+^ cells present in the same field of view at days 0, 1, 2, 4, 7, 14, and 21. Each point represents one mouse. Note the reversal of the correlation between D4 and D21 (*n* = 5 mice for each time and each condition). **(D)** Reverse transcription–qPCR quantifications of proinflammatory gene expression (TNF-α, CD86, CD32 CD16, NOS2, and IL-6), anti-inflammatory gene expression (TGF-β, IGF-1, Arg1, CD206, and IL-10), and neutrophil marker (Ly6G) gene expression, respectively, 7 and 14 days after LPC incubation when normalized to the gene expressions observed on D4. Asterisk indicate statistical significance *p* < 0.05 (*n* = 3 mice for each time). **(E)** Graphs representing the number of cells in subregions of the ipsilateral spinal cord and the corresponding number of axons counted in the same regions as a function of day after LPC incubation. Regions are defined on the anatomical scheme. Asterisk indicate statistical significance *p* < 0.05; two asterisks indicate statistical significance *p* < 0.01 (we used at least five mice for each condition and for each time; total number *n* = 22).

Demyelination has been associated with the activation of phagocytic inflammatory cells (Chu et al., 2019; McMurran et al., 2019). Whether these inflammatory cells contribute to the degenerative processes was investigated by the application of our demyelination model on Thy1–CFP//LysM-EGFP mice. In these mice, monocytes, granulocytes, and macrophages are labeled with EGFP throughout their lifetime in the CNS unless they differentiate into monocyte-derived dendritic cells (Caravagna et al., 2018). We thus quantified in real time the density of these inflammatory cells in the dorsal spinal cord and found that inflammation is significantly increased 4 days after LPC incubation (Figures 4A,B). A significant reduction was then transiently observed on D7 prior to a large second accumulation of GFP^+^ cells that peaked on D14 and completely resorbed by D28, time of full axonal recovery (Figure 4B). Whereas inflammatory response presented significant intersubject variability, we found at all time points that the axonal densities observed in every subject correlated linearly with the corresponding GFP^+^ cell densities (Figure 4C). Noteworthy, though, axonal densities were all the more preserved in animals for which GFP^+^ cell densities were low during the first week; axons were all the more regenerated in animals for which GFP^+^ cell densities were high when considering the second and third week (Figure 4C). These results therefore suggested that the two peaks of GFP^+^ cell densities observed, respectively, on D4 and D14 (Figure 4B) corresponded to inflammatory responses with different functional phenotypes.

To further characterize this phenotypic switch, we tested by RT-qPCR on GFP^+^ FACS-sorted cells the expression of six proinflammatory genes [tumor necrosis factor α (TNF-α), CD86, CD32 CD16, NOS2, and interleukin 6 (IL-6)] and five anti-inflammatory genes [transforming growth factor (TGF-β), IGF-1, Arg1, CD206, and IL-10], as well the expression of Ly6G as a marker of granulocytes and neutrophils. Proinflammatory genes (Figure 4D, red squares) presented a maximal transcription on D7, followed by a significant decline at D14 in particular for TNF-α, CD32 CD16. A significant and continuous decrease over time was instead observed for NOS2 and IL-6. Conversely anti-inflammatory genes presented a systematic increase over time (Figure 4D, green squares), whereas Ly6G expression declined over time (Figure 4D, yellow square).

The idea of an inflammatory switch was further supported by the existence of two different patterned distributions of cells during the first week (Figure 4E). GFP^+^ cells mostly spread in the vicinity of the dorsal vein until D4 despite a location of the lesion site close to the dorsal root. On D7, however, most GFP^+^ cells were observed close to the dorsal root, to finally distribute themselves equally all over the ipsilateral spinal surface. Interestingly, axon losses between D2 and D4, the peak of GFP^+^ infiltration, were all the more important close to the dorsal vein where cells were located. Conversely, the percentage of regenerated axons between D7 and D14 was more important on the side of the dorsal root where GFP^+^ cell density was highest (Figure 4E).

## Discussion

Lysophosphatidylcholine model of demyelination is among the most used model for studying demyelination, the myelin repair, and the way to improve it. So far, LPC was always administered into the brain or spinal cord parenchyma by capillary injection (Hall, 1972; Arnett et al., 2004; McMurran et al., 2019). Localized axonal degeneration was thus mainly attributed to mechanical lesion by injection pipette and hence LPC effect considered as specific to myelin and OLs. In this study, we have developed an LPC model without mechanical injection and monitored cellular interactions at the lesion site *in vivo* over time. Implantation of a unique dorsal glass window on multicolor fluorescent mice (Fenrich et al., 2012) offered longitudinal imaging access to axonal networks, myelin sheath, and immune cells after LPC lesion. Thanks to simultaneous two-photon and CARS microscopies, we evidenced that LPC that initially targets OL is also responsible for axonal degeneration.

We already showed in fixed tissue that CARS microscopy is beneficial for detecting the early changes of myelin in an experimental autoimmune encephalomyelitis (EAE) model of demyelination (Gasecka et al., 2017). We have here implemented intravital CARS microscopy and further showed that the dynamics of myelin coverage contains crucial information to elucidate the mechanism by which LPC triggers axon degeneration as early as D0. This axonal degeneration was unlikely because of direct action of LPC because neurotoxicity was not reported in culture (Vereyken et al., 2009) and because Thy1–CFP^+^ axons in the imaged zones are in fact anatomically shielded from extracellular fluids by OLs *in vivo*. Time-coded images of the demyelinating events indicated a clear coincidence between the chemically induced insults to the myelin sheath and the changes in axonal morphology. The mechanical constraints exerted by OLs on axons likely explained the formation of spring-like axonal shapes and subsequent Wallerian degeneration of axons. Degeneration indeed started in the first few hours after exposition and progressed in a two-step process during the following 4 days.

Myelosomes detection by CARS microscopy (Gasecka et al., 2017) contributed to the overall CARS intensity until their complete elimination from the parenchyma. Thus, at late degenerative stages, the strong CARS background was likely explained by the lipid spreading from the degenerated membranes of these myelosomes. As a result, an apparent uncoupling between the kinetics of CARS signal and the kinetics of axonal losses in the lesioned area was observed until D4. Whereas both axonal density and CARS signal recovered to prelesion values within 4 weeks, a similar decorrelation was observed during the recovery phase of these two parameters: recovery was early but slow for axonal density whose recovery started on D7; it was delayed but rapid for CARS signal whose recovery started on D14. Such delayed recovery of the CARS signal might be explained by the requirement for proliferation, recruitment, and differentiation of OPC to replace apoptotic OLs whose GPR17 receptor had been activated (Mayo et al., 2012; Seyedsadr and Ineichen, 2017). In the case of neurons instead, recruitment of progenitor cells is unlikely (Mothe and Tator, 2005). The fact that axons regenerated so early after LPC lesion therefore suggested that neuronal death was probably not involved and that the regenerative axonal sprouting instead occurred as soon as their oligodendroglial and inflammatory environment stopped from being deleterious.

Individual axon imaging confirmed that axonal regeneration started between D4 and D7, whereas, at D7, myelin coverage remaining at the minimal lipid density was present in the environment. Indeed, the ratio of myelinated axons was transiently low at D7, and it increases at D14. Our results therefore confirmed that the regenerative processes were differently and independently regulated by the postlesion physicochemical environment in the case of neurons and OLs. Different chemokines (Balabanov et al., 2007) and cytokines (Cannella and Raine, 2004; Ramesh et al., 2012) are released in the environment at the first sign of OLs suffering. These participate to the activation of microglia and astrocytes and subsequent recruitment of innate immune cells (Domingues et al., 2016). These immune cells can be harmful through the disruption of glutamate hemostasis or the production of nitric oxide and reactive oxygen species (Peferoen et al., 2014; Domingues et al., 2016). Yet they can also have a beneficial role through the release of TGF-b and IL-10, which are anti-inflammatory and prohealing growth factors (Peferoen et al., 2014).

Our results outlined two waves of innate immune cell infiltrations with different phenotypes, the first between D2 and D4 and the second between D7 and D14. The negative correlation between axons and innate immune cell densities observed on D4, as well as the significant secondary decrease of axon density between D2 and D4, supported the idea that the first wave of infiltration could be harmful to the axons. Similar conclusion was indeed made in an EAE model of inflammation where the initial infiltration of EGFP^+^ neutrophils triggered the fast degeneration of axons (Caravagna et al., 2018). On the other hand, the significant positive correlation between axons and innate immune cell densities observed among mice on D14, as well as the significant increases of axons between D7 and D14, supported the idea that the second wave of EGFP^+^ cell infiltration could be beneficial for axonal regeneration. Noteworthy, this second wave was coincident with the fast recovery of CARS signal, as expected from a beneficial impact on remyelination, and despite the only weak impact observed on demyelination for the first wave. As the clearance of myelin debris is a prerequisite for remyelination (Peferoen et al., 2014), we propose that the second wave is responsible for an amplification of the environment cleaning and therefore mainly composed of phagocytic immune cells with rather anti-inflammatory phenotype.

Two characteristic distribution patterns were evidenced for these two waves supporting the existence of at least two different subpopulations. Indeed, EGFP^+^ cells of the first wave expressed *Nos2* and *IL6* significantly more than EGFP^+^ cells at D14, and they were significantly more present medially next to the dorsal spinal vein despite a lateral location of the lesion. However, cells of the second wave expressed significantly more anti-inflammatory genes such as *Tgf-beta*, *Igf1*, *Arg1 cd206*, and *IL10*, and they were preferentially found on the DRG side and progressively invaded the lesion. A DRG resident pool of macrophages (Krishnan et al., 2018) was likely recruited in a second stage, while initial inflammatory response was led by vascular neutrophils (Neirinckx et al., 2014).

## Conclusion

In conclusion, this study shows that LPC exposition can itself cause axon degeneration first as a consequence of oligodendroglial suffering and second due to innate immune cell infiltration. It emphasizes the importance of multimodal nonlinear optical microscopies to characterize the subcellular substrate of myelin plasticity. By pointing out the crucial role of dynamic inflammatory processes, it highlights the requirement of intravital studies to unravel multicomponents physiological response involved in neurodegenerative diseases. On the same animal over time, it finally illustrates the ambivalence of innate immune responses, thereby paving the way to immunomodulatory therapies as a strategy to improve assistance to neurodegenerative patients.

## Data Availability Statement

All datasets generated for this study are included in the article/Supplementary Material.

## Ethics Statement

The animal study was reviewed and approved by the Comité d’éthique à l’expérimentation animale no 71.

## Author Contributions

FD and BE designed the animal model, the imaging experiments, interpreted the results, and wrote the manuscript with inputs of all the authors. SB aligned and maintained the multimodal microscope. All authors contributed to image acquisition. BE, EB, and CK did the experiments, analyzed the images, and quantified all the data.

## Conflict of Interest

The authors declare that the research was conducted in the absence of any commercial or financial relationships that could be construed as a potential conflict of interest.

## Supplementary Material

FIGURE S1 Comparison of MBP immunolabeling and CARS microscopy. **(A)** CARS signal detection and MBP Immunolabeling on the same coronal sections of the spinal cord at LPC incubation site on D7. Four rectangle areas (dotted lines) were defined on each section: 1, 2, and 3 are outside the LPC lesion while 4 is inside the LPC lesion. **(B)** Graph showing the positive correlation between CARS and MBP signal intensities (*n* = 3 mice). **(C)** High magnification image in the white matter of the dorsal spinal cord. Scale bars represent 50 μm for **(A)** and 10 μm for **(C)**.

FIGURE S2 Longitudinal imaging of the fate of individual myelinated axons after LPC incubation. **(A)** CARS and Thy1–CFP signals collected from the same area over 14 days showing individual axon bundle at D0. Note that axonal degeneration was faster and more extensive than the loss of CARS signal. Axonal regeneration was already significant on D7 when myelin coverage was still minimal. Myelination was restored by D14. **(B)** High magnification of the white square in the **(A)**. Scale bars represent 10 μm. **(C)** Multicolor images of the mouse presented in Figure 2 showing the superimposition of the following channels: CARS signal (Purple), Thy1–CFP^+^ axons (Blue), and LysM-GFP^+^ cells (Green) 7, 14, and 21 days after LPC incubation.
