# Supplementary figures and images for: Longitudinal Intravital Microscopy Reveals Axon Degeneration Concomitant With Inflammatory Cell Infiltration in an LPC Model of Demyelination
Source: Front Cell Neurosci. 2020 Jun 23;14:165. doi: 10.3389/fncel.2020.00165 (PMC7324938; doi:10.3389/fncel.2020.00165)

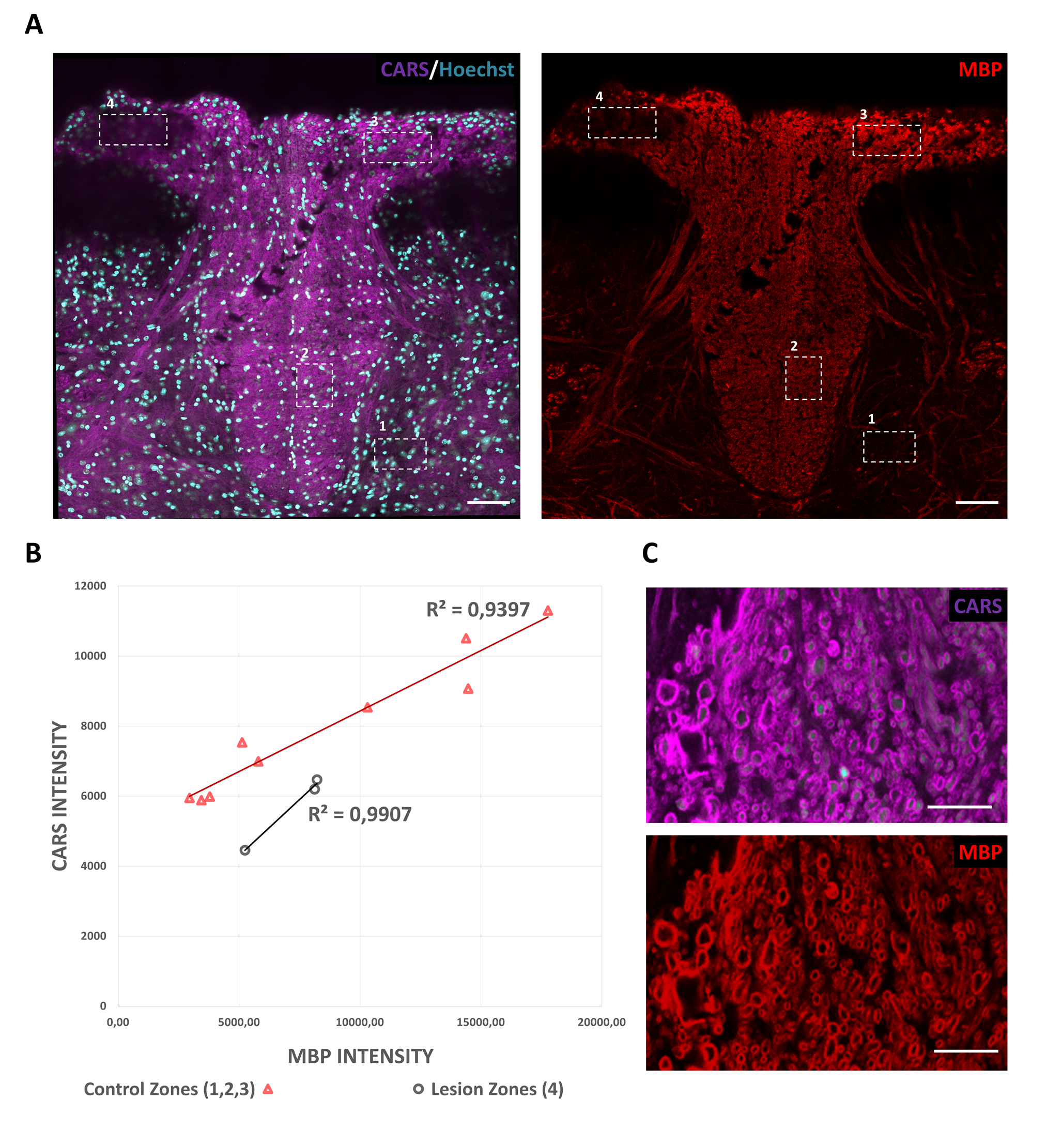

Supplement: FIGURE S1 — Comparison of MBP immunolabeling and CARS microscopy. (A) CARS signal detection and MBP Immunolabeling on the same coronal sections of the spinal cord at LPC incubation site on D7. Four rectangle areas (dotted lines) were defined on each section: 1, 2, and 3 are outside the LPC lesion while 4 is inside the LPC lesion. (B) Graph showing the positive correlation between CARS and MBP signal intensities (n = 3 mice). (C) High magnification image in the white matter of the dorsal spinal cord. Scale bars represent 50 μm for (A) and 10 μm for (C). [file Image_1.tif]

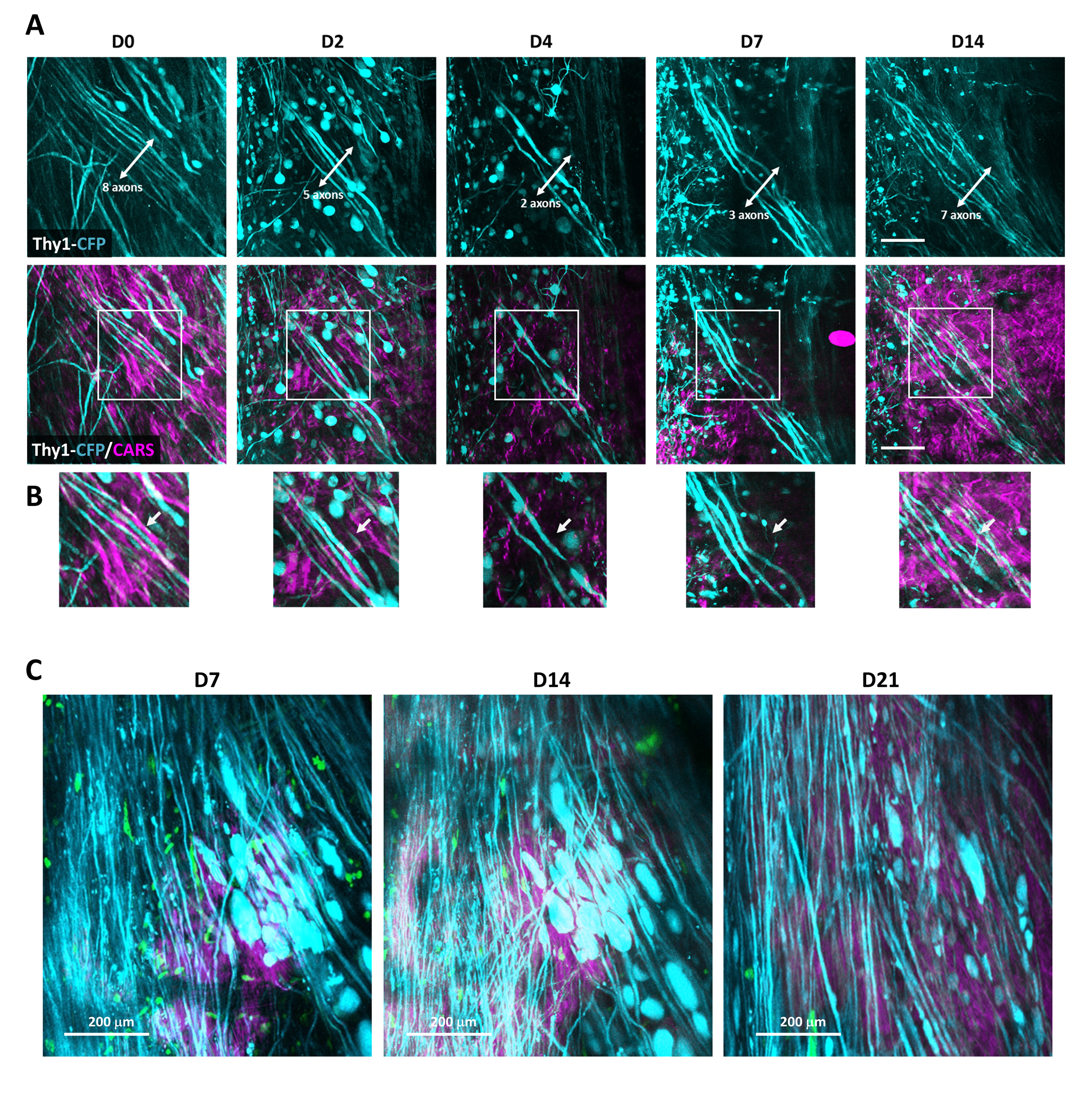

Supplement: FIGURE S2 — Longitudinal imaging of the fate of individual myelinated axons after LPC incubation. (A) CARS and Thy1–CFP signals collected from the same area over 14 days showing individual axon bundle at D0. Note that axonal degeneration was faster and more extensive than the loss of CARS signal. Axonal regeneration was already significant on D7 when myelin coverage was still minimal. Myelination was restored by D14. (B) High magnification of the white square in the (A). Scale bars represent 10 μm. (C) Multicolor images of the mouse presented in Figure 2 showing the superimposition of the following channels: CARS signal (Purple), Thy1–CFP+ axons (Blue), and LysM-GFP+ cells (Green) 7, 14, and 21 days after LPC incubation. [file Image_2.tif]
